# Supplementary material for: Hydrogen Peroxide Promotes the Production of Radiation-Derived EVs Containing Mitochondrial Proteins
Source: Antioxidants (Basel). 2022 Oct 27;11(11):2119. doi: 10.3390/antiox11112119 (PMC9686922; doi:10.3390/antiox11112119)
Supplement: Supplementary file 1 [file antioxidants-11-02119-s001.zip › antioxidants-1915012-supplementary.pdf]

Supplementary Figures:
Hydrogen Peroxide Promotes the Production of Radiation-Derived EVs Containing Mitochondria

Caitlin Miller et al.

Cell Line
Authentication Service
STR Profile Report

FTA Barcode: STRC0621

ATCC Sales Order: SO1232033

Test Results for Submitted Sample					ATCC Reference Database Profile			
**Locus**	**Query Profile: PC-3/Clone 695**				**Database Profile: PC-3; Prostate Adenocarcinoma; Human (Homo sapiens)**			
D3S1358	16							
TH01	6	7			6	7		
D21S11	29							
D18S51	14	15						
Penta_E	10	17						
D5S818	13				13			
D13S317	11				11			
D7S820	8	11			8	11		
D16S539	11				11			
CSF1PO	11				11			
Penta_D	9							
Amelogenin	X				X			
vWA	17				17			
D8S1179	13							
TPOX	8	9			8	9		
FGA	24							
D19S433	14							
D2S1338	18	20						
Number of shared alleles between query sample and database profile:								12
Total number of alleles in the database profile:								12
Percent match between the submitted sample and the database profile:								100

The allele match algorithm compares the 8 core loci plus amelogenin only, even though alleles from all loci will be reported when available.

NOTE: *Loci highlighted in grey (8 core STR loci plus Amelogenin) can be made public to verify cell identity. In order to protect the identity of the donor,* ***please do not publish*** *the allele calls from all the STR loci tested.*
Electropherograms showing raw data are attached.

Explanation of Test Results
Cell lines with 80% match are considered to be related; i.e., derived from a common ancestry. Cell lines with between a 55% to 80% match require further profiling for authentication of relatedness.

[ ] The submitted sample profile is human, but not a match for any profile in the ATCC STR database.

[X] The submitted profile is an exact match for the following ATCC human cell line(s) in the ATCC STR database (8 core loci plus Amelogenin): CRL-1435

[ ] The submitted profile is similar to the following ATCC human cell line(s):

[ ] An STR profile could not be generated.

Additional Comments:

n/a

Supplementary Figure S1: Cell line authentication (Clone 695)

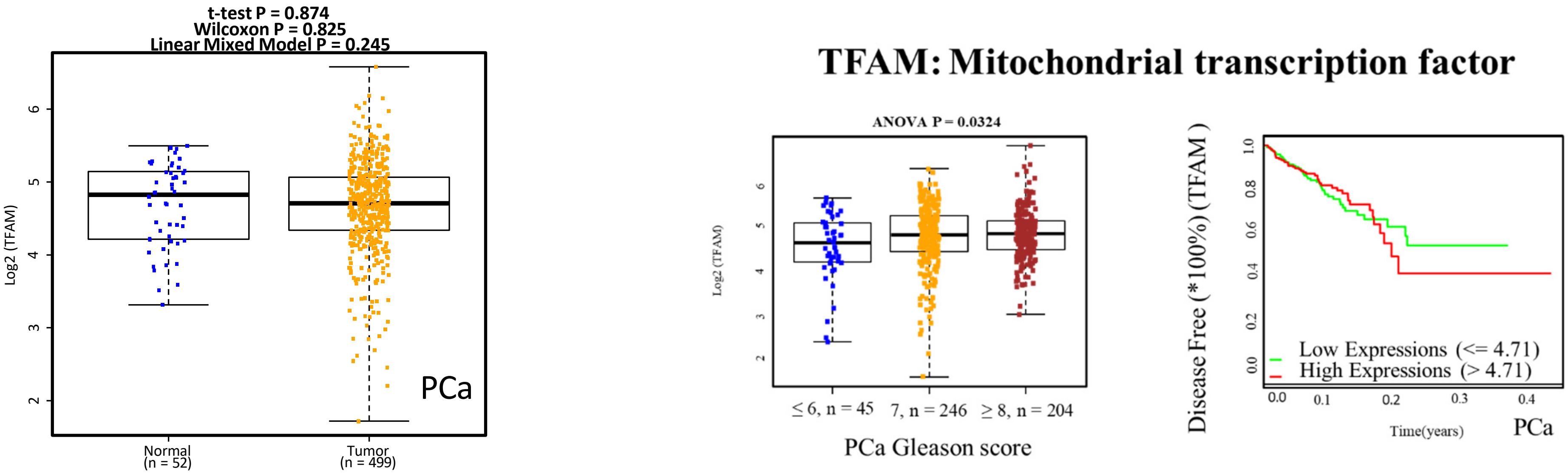

Supplementary Figure S2. Oncomine TCGA analysis of PCa patients in varying stages of tumor progression. TFAM mRNA expression at varying Gleason scores and Disease free percentage of patients with TFAM mRNA expression cut off at 4.71

A. Imaris Algorithm for mitochondrial segmentation
Enable Region Of Interest = false
Enable Region Growing = true
Enable Tracking = false
Enable Classify = true
Enable Shortest Distance = false
[Source Channel]
Source Channel Index = 2
Enable Smooth = true
Surface Grain Size = 0.153 µm
Enable Eliminate Background = false
Diameter Of Largest Sphere = 0.572 µm
[Threshold]
Enable Automatic Threshold = false
Manual Threshold Value = 123.967
Active Threshold = true
Enable Automatic Threshold B = true
Manual Threshold Value B = 576.546
Active Threshold B = false
Region Growing Estimated Diameter = 0.200 µm
[Filter Seed Points]
"Quality" above 10.2
[Filter Surfaces]
"Number of Voxels Img=1" above 10.0

B. Segmented Mitochondrion

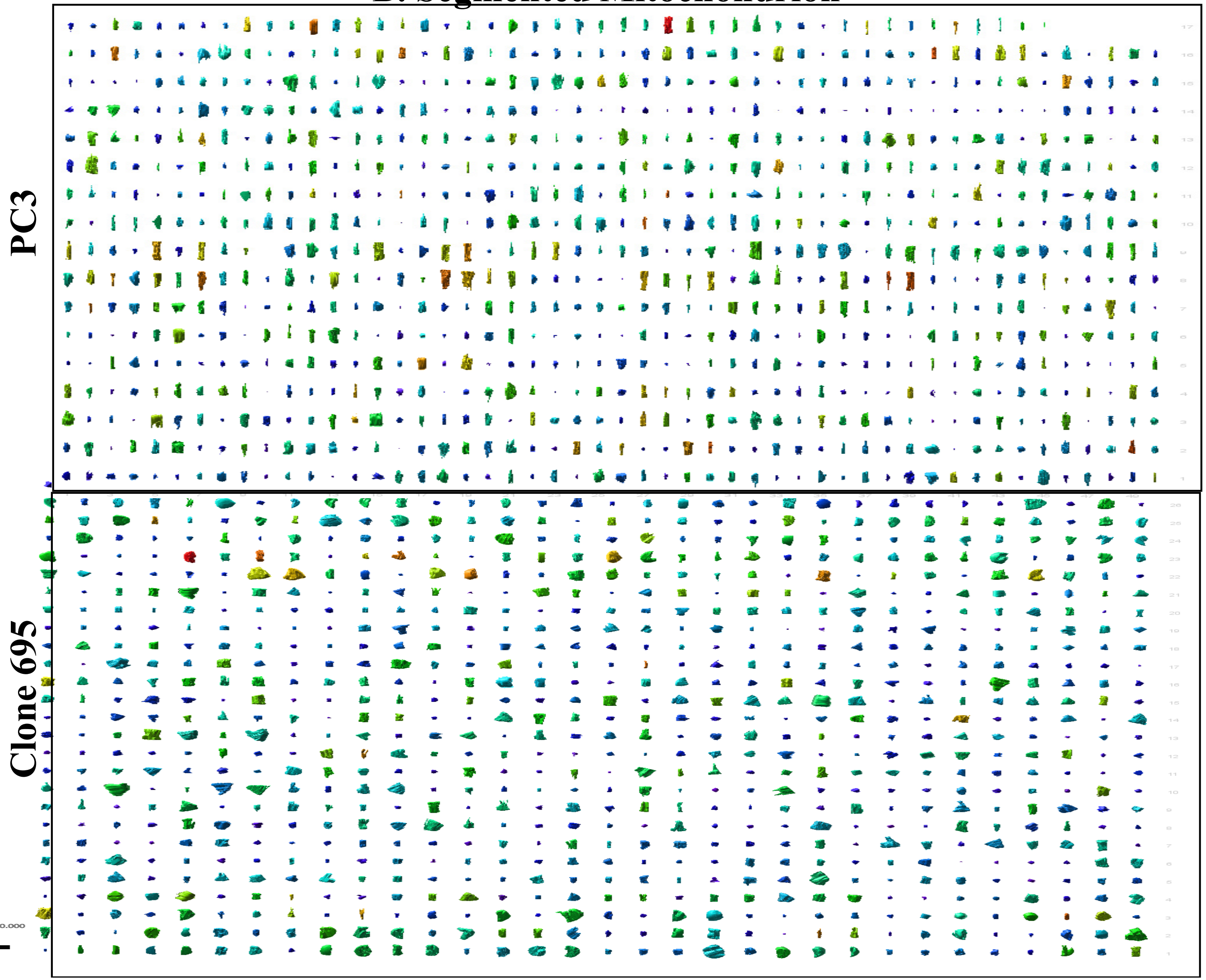

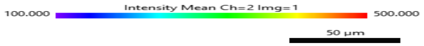

Supplemental Figure S3: A) Automated algorithm generated by Imaris software for segmentation of mitochondrion. B) Individual segmented mitochondrion.

A. Mitochondrial Proteins in RT-derived EVs

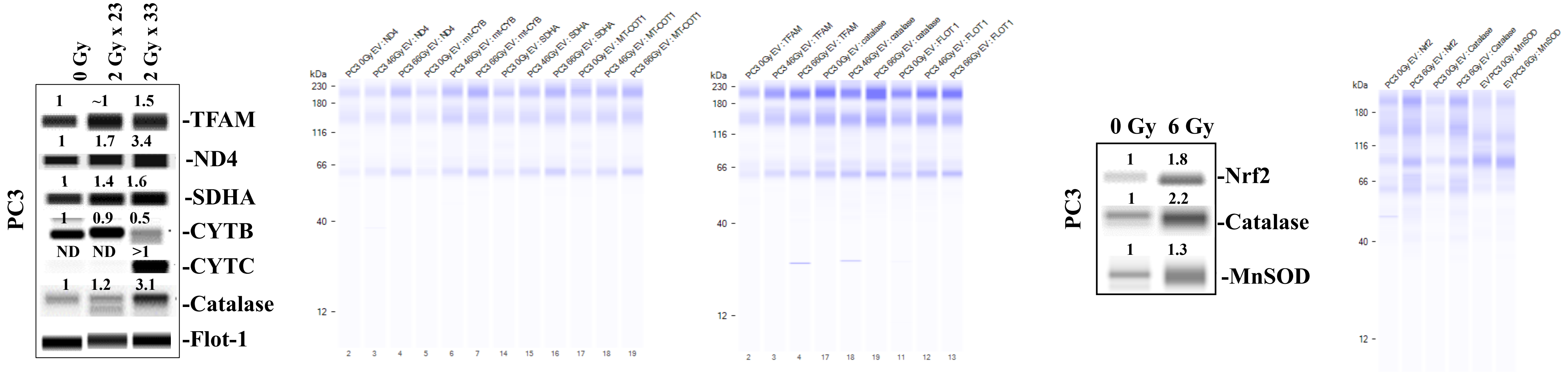

B. Total loading proteins (1ug/ul) in RT-derived EVs and H_2O_2-derived EVs

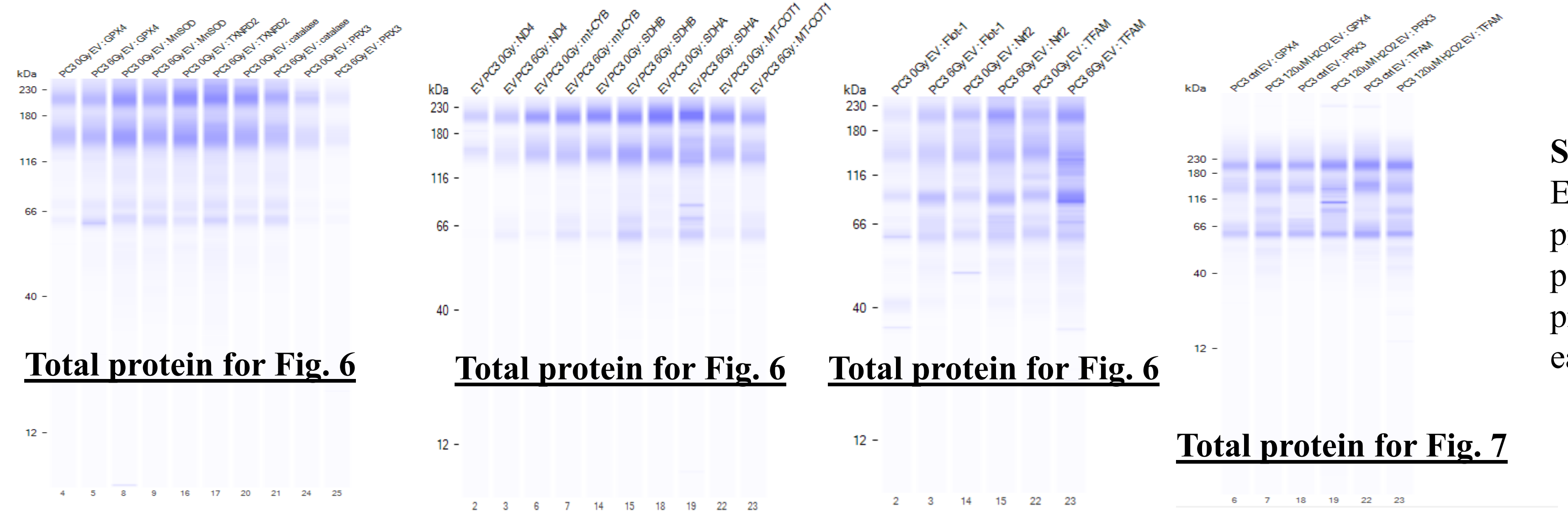

Supplemental Figure S4: A) Expressions of mitochondrial proteins in EVs isolated from post-RT PC3 cells. B) Total protein loading (1ug/ul) of each representative sample.
